# Bazedoxifene Regulates Th17 Immune Response to Ameliorate Experimental Autoimmune myocarditis via Inhibition of STAT3 Activation

**DOI:** 10.3389/fphar.2020.613160

**Published:** 2021-02-10

**Authors:** Jing Wang, Tianshu Liu, Xiongwen Chen, Qiaofeng Jin, Yihan Chen, Li Zhang, Zhengyang Han, Dandan Chen, Yuman Li, Qing Lv, Mingxing Xie

**Affiliations:** ^1^ Department of Ultrasound, Union Hospital, Tongji Medical College, Huazhong University of Science and Technology, Wuhan, China; ^2^ Hubei Province Key Laboratory of Molecular Imaging, Wuhan, China; ^3^ Cardiovascular Research Center and Department of Physiology, Temple University School of Medicine, Philadelphia, PA, United States

**Keywords:** Th17 cell, interleukin-6, signal transducer and activator of transcription 3, experimental autoimmune myocarditis, autophagy

## Abstract

Myocarditis is a type of inflammatory cardiomyopathy that has no specific treatment. Accumulating evidence suggests that Th17 cells play a prominent role in the pathogenesis of myocarditis. Interleukin-(IL)-6-mediated signal transducer and activation of transcription 3 (STAT3) signaling is essential for Th17 cell differentiation and secretion of inflammatory cytokines. Bazedoxifene inhibits IL-6/STAT3 signaling in cancer cells, but its effect on the Th17 immune response induced by myocarditis remains unknown. Here we explore the effect of Bazedoxifene on Th17 immune response and cardiac inflammation in a mouse model of experimental autoimmune myocarditis, which has been used to mimic human inflammatory heart disease. After eliciting an immune response, we found Bazedoxifene ameliorated cardiac inflammatory injury and dysfunction. Th17 cells and related inflammatory factors in splenic CD4^+^ T cells at day 14 and in the heart at day 21 were increased, which were reduced by Bazedoxifene. Furthermore, Bazedoxifene could regulate autophagy induction in polarized Th17 cells. In conclusion, Bazedoxifene affected STAT3 signaling and prevented cardiac inflammation deterioration, so may provide a promising therapeutic strategy for the treatment of experimental autoimmune myocarditis (EAM).

## Introduction

Myocarditis is an inflammatory cardiomyopathy that leads to acute heart failure, dilated cardiomyopathy, and sudden death (Bracamonte-Baran and Cihakova, 2017). Current treatments for myocarditis include supportive therapy and immunosuppressive therapy, but the effectiveness of these treatments is not yet clear (Jensen and Marchant, 2016). Hence, there is a need to further understand the mechanism of myocarditis and explore novel pharmacologic treatments in order to ameliorate cardiac injury and help patients to survive.

The immune response involved in the progression of experimental autoimmune myocarditis (EAM) is complex. Studies have suggested a critical role for CD4^+^ T cells in the development of EAM (Błyszczuk, 2019). The conventional view is that autoimmune diseases are primarily dependent on Th1 and Th2 cells (Nindl et al., 2012). Recently, Th17 cells have been identified as playing a crucial role in this process (Maddur et al., 2012). Th17 cells increase immune effects in various inflammatory and autoimmune diseases by secreting proinflammatory cytokines, including IL-17A, IL-21, and IL-22 (Miossec et al., 2009). Regulatory T cells (Treg) restrain excessive responses of Th17 cells by producing immunosuppressive cytokines, including IL-10, IL-35, and TGF-β (Josefowicz et al., 2012). IL-6 is essential for the differentiation of naive T cells into Th17 cells by activating retinoid-related orphan receptor gamma t (RORγt), a specific transcriptional regulator, downstream of the signal transducer and activator of transcription 3 (STAT3) (Ivanov et al., 2006; Yang et al., 2007). An imbalance between Th17 and Treg cells has been reported to be associated with EAM progression (Cheng et al., 2016). Therefore, a therapeutic strategy might be to attenuate the excessive immune response of Th17 cell by inhibiting STAT3 activation.

Bazedoxifene is a third-generation estrogen receptor modulator that has been approved by the FDA (Food and Drug Administration) for prevention and treatment of osteoporosis (Bueno et al., 2017). Recent evidence indicates that using multiple ligand simultaneous docking (MLSD) and drug repositioning approaches, BAZ exhibits a new function targeting the IL-6/GP130 protein-protein interface (Li et al., 2014). Bazedoxifene can attenuate HCT-15 and HEPG2 xenograft tumor burden by inhibiting STAT3 activation induced by IL-6 (Ma et al., 2019; Wei et al., 2019). However, its therapeutic potential in EAM has not been addressed. The molecular mechanism of the IL-6/STAT3 signaling pathway and its effect on Th17 cell development in EAM needs further exploration.

## Materials and Methods

### Animals

Male BALB/c mice aged 6–7 weeks (weighing 18–20 g) were purchased from HFK Bioscience CO., LTD. (Beijing, China). All animal experiments were performed in accordance with the Institutional Animal Use and Care Committee of Tongji Medical College, Huazhong University of Science and Technology (Wuhan, China).

### EAM Induction and Bazedoxifene Treatment *in vivo*

Mice were randomly assigned to three groups: 1) control group, 2) EAM group, and 3) EAM + Bazedoxifene (hereinafter referred as BAZ) group. Mice were immunized with cardiac-specific peptide (MHC-α_614–629_: Acetyl-SLKLMATLFSTYAS) purchased from GL Biochem (Shanghai) Ltd. The peptide was dissolved in saline and emulsified with complete Freund’s adjuvant (CFA) in a 1:1 ratio. 200 μg peptide in 0.2 ml of the emulsion was injected subcutaneously into one side of the axillary and inguinal lymph node region on day 0, and the same dosing was injected subcutaneously into the opposite side on day 7. Bazedoxifene (5 mg/kg) was administrated in mice via intragastric gavage daily from day 7 until sacrifice at day 14 or day 21. Mice infused with saline and treated with a vehicle served as controls.

### Echocardiography

Transthoracic echocardiography of mice was performed at day 21 using the Vevo 1,100 instrument (Visual sonics, Toronto, Canada) with transducer MS400 (30 MHz). To induce sedation and immobility before echocardiography, mice were anesthetized with 2% sodium pentobarbital at 80 mg/kg. After a two-dimensional parasternal short-axis image of the left ventricle at the level of the papillary muscles obtained in end-diastole, two-dimension guided M-mode images were acquired. The left ventricular ejection fraction (EF) and fractional shortening (FS) were calculated using the Vevo 1,100 software.

### Measurement of Systolic Blood Pressure

The Softron BP-2010 Series, a noninvasive tail-cuff system, was utilized for the measurement of Systolic Blood Pressure (SBP), Mean Blood Pressure (MBP), and Diastolic Blood Pressure (DBP). All mice were first trained to stay quietly in a restrainer placed on a warm pad for a period of 15 min before the measurement.

### Cardiac Infiltrating Cell Isolation

Cardiac-infiltrating cells were isolated from EAM hearts using some minor modifications of the method described previously (Alexander et al., 2013). In brief, hearts were collected and perfused with HEPES buffer (20 mM HEPES, 130 mM NaCl, 3 mM KCl, 1 mM NaH_2_PO_4_, 4.5 mM glucose, pH = 7.4) and then minced and digested in 0.1% collagenase B solution (C6885, Sigma, United States; 1 mg/ml in HEPES buffer) at 37 °C. The digested cell suspension was filtered through 40 μm cell strainers to prepare single-cell suspension. The cardiac lymphocytes isolated by density gradient centrifugation were cultured in complete RPMI-1640 medium with 10% fetal bovine serum.

### Histopathology and Immunohistochemistry

Paraffin-embedded heart sections were first dewaxed and hydrated through an ethanol gradient and used for hematoxylin and eosin (H and E) or Masson’s trichrome staining. The inflammation score was as follows: grade 0, no inflammatory infiltrates; grade 1, <25% of a cross-section involved; grade 2, 25–50% of a cross-section involved; grade 3, 50–75% of a cross-section involved; >75% of a cross section involved (Quah et al., 2007). For immunostaining, after antigen retrieval, all slides were incubated in H_2_O_2_ to block endogenous peroxidase activity and later in BSA (5%) to block nonspecific antigen binding sites. This was followed by incubation with primary anti-CD45 (#70257, CST) or anti-IL-6 antibody (#D220828, Sangon, Shanghai, China) overnight at 4 °C and subsequent treatment with horse radish peroxidase-conjugated secondary antibody for 1 h at 4 °C according to manufacturer’s protocols.

### Th17 Induction *in vitro*

Naïve CD4^+^ T cells were sorted from the splenocytes of healthy BALB/c mice using a Naïve CD4^+^ T cell isolation kit (Miltenyi Biotec MACS, Germany). For detection Th17 differentiation and proliferation, cells were cultured with or without 5 μM carboxyfluorescein succinimidyl ester (CFSE) labeling according to the manufacturer’s protocols (Livak and Schmittgen, 2001). The cultured condition of cells after sorting was IMDM medium comprised of 10% heat inactivated FBS (Gibco, United States), 2 mM of l-glutamine (Sigma), 0.1 mM nonessential amino acids (Sigma), 1% penicillin/streptomycin, and 100 µM β-mercaptoethanol (Sigma-Aldrich). For Th17 cells’ differentiation, anti-mouse IFN-γ (10 μg/ml; Biolegend, United States), anti-mouse IL-4 (10 μg/ml; Biolegend, United States), recombinant mouse IL-6 (60 ng/ml; Biolegend, United States), and recombinant mouse TGF-β1 (5 ng/ml; Biolegend, United States) were added in 96-well plates with plate-bound anti-mouse CD3 (5 μg/ml; Biolegend, United States) and anti-mouse CD28 (5 μg/ml; Biolegend, United States). After 72 h, cells were collected for further experiments.

### Flow Cytometric Analysis

For Treg detection, cells were first stained with FITC-conjugated anti-mouse CD45 (BD, United States), PE-CY-7-conjugated anti-mouse CD4 (BD, United States), and APC-conjugated anti-mouse CD25 (BD, United States). For Th17 cell detection, cells were stimulated with Cell Stimulation Cocktail in culture medium for 4 h before surface staining using FITC-conjugated anti-mouse CD45 and PE-CY-7-conjugated anti-mouse CD4. After surface staining, cells were fixed and permeabilized with fixation/permeabilization buffer, followed by staining with PE-conjugated anti-mouse Foxp3 (eBIOSCIENCE, United States) for Tregs and PE-conjugated anti-mouse IL-17A (BD, United States) for Th17 cells. For dendritic cells detection, cells were stained with FITC-conjugated anti-mouse CD11c (BD, United States). Cells were analyzed on a FACS flow cytometer (CytoFLEX, Beckman, United States and BD Biosciences, United States).

### Real-Time Quantitative RT-PCR Analysis

Total RNA was extracted using Trizol® reagent (Invitrogen) and converted into cDNA using PrimeScript RT Reagent Kit (Takara Biotechnology). Real-time qRT-PCR analyses were performed on a Bio-rad CXF CONNECT Detector system using SYBR green master mix (Takara Biotechnology), and relative expression was calculated using the CT method (Xiao et al., 2017). β-actin was used as internal reference and the 2^ΔΔCT^ method was used for data analysis.

### Western Blotting Analysis

Protein extracts of cells or tissue were prepared in lysis buffer containing a protease inhibitor and phosphatase inhibitor. Proteins were resolved on 10% sodium dodecyl sulfate-polyacrylamide gel electrophoresis (SDS-PAGE) gels and then immunoblotted to nitrocellulose membranes. After being blocked in Tris buffered saline with Tween 20 with 5% skim-milk for 1 h at RT, the membranes were incubated with primary antibodies against P-STAT3 (Tyr705), STAT3, Foxp3, RORγt, beclin-1, LC3 I/II, P62, or β-actin at 4 °C overnight, and then washed and further incubated with secondary HRP-conjugated antibody for 2 h at room temperature. The membranes were finally washed and the blots were developed with enhanced chemiluminescence kit (Pierce). The levels of target proteins were normalized to β-actin.

### Measurement of Cytokines by ELISA

For the enzyme-linked immunosorbent assay (ELISA), serum samples were collected by cardiac puncture from each group of mice. The levels of IL-23, IL-6, and IL-17A were quantified using ELISA kits (Boster Biological Technology, Wuhan, China) according to the manufacturer’s instructions.

### Transmission Electron Microscopy

The Th17 polarized cells were fixed with 2.0% glutaraldehyde in 0.1 M sodium cacodylate buffer, pH 7.4, and then post-fixed in 1% osmium tetroxide, dehydrated in ethanol, and embedded in epon. Ultrathin sections of cells were collected on formvar-coated grids and were stained with uranil acetate and lead citrate. The samples were examined with a FEI Tecnai G^2^ 20TWIN Transmission Electron Microscope operated at 200 KV.

### Confocal Microscopy and Immunostaining

The Th17 polarized cells were obtained after 72 h incubation and fixed with paraformaldehyde at RT for 15 min. After permeabilization and blockage with PBS buffer containing 0.1% TritonX-100 and 0.5% normal goat serum at RT for 1 h, cells were stained using FITC-, Cy3-, and PE-Cy-5.5- antibodies to mouse P-STAT3 (Tyr705), beclin-1, LC3 I/II, and P62. Images were captured with a laser-scanning confocal microscope (LSM 880, Carl Zeiss, Germany) by an observer who was blinded to the identities of the samples.

### Statistical Analysis

GraphPad Prism software (version 5.0 for Windows, San Diego, CA, United States) was used for all statistical analyses. Statistical analysis was performed using the unpaired Student’s t test for two groups and one-way ANOVA analysis for multiple comparisons. All values were presented as mean ± SEM. Statistical significance was accepted at *p* values < 0.05.

## Results

### Bazedoxifene Ameliorated Cardiac Inflammation and Injury Induced by EAM

**FIGURE 1:**
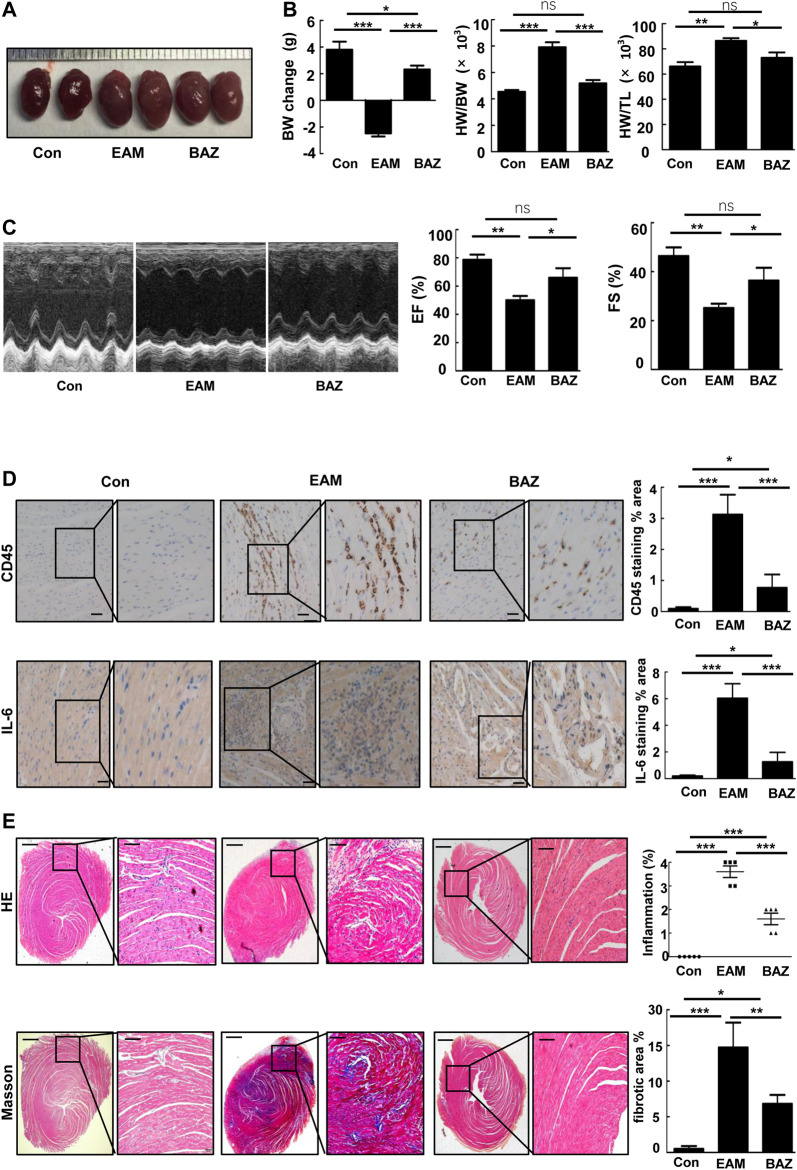
Bazedoxifene ameliorated cardiac inflammation and injury induced by EAM and improved EAM-induced left ventricular dysfunction **(A)**. Representative heart images of the indicated groups **(B)**. Change in body weight, HW/BW ratio, and HW/TL ratio of the indicated groups **(C)**. Representative echocardiography M-mode images and analysis of left ventricular function. Representative images of immunohistochemistry of inflammatory cells stained with anti-CD45 and anti-IL-6 **(D)** in each heart section. Scale bars: 50 μm. E and **F**. Representative H and E-stained and Masson staining images of left ventricular sections of indicated groups. Bar graphs on the right show quantitation of data **(E)** (*n* = 5). Scale bars: left 0.5 mm, inset 100 μm. Scale bars: left 0.5 mm, inset 100 μm *p < 0.05, **p < 0.01, ***p < 0.001.

Compared with untreated EAM mice, Bazedoxifene-treated mice exhibited less severe EAM, including less reduction in body weight, reduced ratios of heart weight/body weight (HW/BW) and heart weight/tibia length (HW/TL), and smaller heart size (Figures 1A,B).

As shown by histopathological analysis, Bazedoxifene decreased inflammatory cell infiltration detected by immunostaining of CD45 (Figure 1D) and IL-6 in the heart tissue of EAM mice (Figure 1D). H&E-stained EAM heart sections demonstrated a high inflammation score, which was reduced by Bazedoxifene treatment (Figure 1E). Myocardial fibrosis was reduced by Bazedoxifene treatment as indicated by reduced collagen deposits in treated groups (Figure 1E).

### Bazedoxifene Reduced EAM-Induced Left Ventricular Dysfunction

Echocardiography was performed to assess cardiac function in each group at day 21 (Figure 1C; Table 1). Cardiac systolic dysfunction, as indicated by left ventricular ejection fraction (EF) and fractional shorting (FS), decreased significantly in the EAM group, whereas these reductions in EF and FS were blunted in the Bazedoxifene-treated group. In addition, blood pressure was measured in control, EAM, and BAZ mice at day 21. As demonstrated in Supplementary Figure S5, there was no significant difference in systolic BP, mean BP, and diastolic BP between different treatment groups (*p* > 0.05).

**TABLE 1:** Echocardiographic analysis of control, EAM and Bazedoxifene treated mice groups.

	Control	EAM	BAZ
Heart rate (bpm)	388 ± 48	419 ± 43	392 ± 30
Ejection fraction%	79 ± 6	50 ± 6.7^aa^	66 ± 13^b^
Fractional shortening%	46 ± 6	25 ± 4^aa^	36 ± 10^b^
LVID diastolic (mm)	3.2 ± 0.4	4.0 ± 0.3^a^	3.4 ± 0.2^b^
LVID systolic (mm)	1.7 ± 0.3	3.0 ± 0.4^aa^	2.19 ± 0.5^b^
LV mass (mg)	46 ± 6	89 ± 16^aa^	64 ± 15^b^

Data are presented as mean ± SEM; LVID, left ventricular internal dimension. ^a^P<0.05; ^aa^ *p* < 0.01; ^aaa^P<0.005 vs. Control. ^b^P<0.05; ^bb^P<0.01; ^bbb^P<0.005 vs. EAM.

### Bazedoxifene Reduced Th17 Cell Infiltration and Inhibited STAT3 Phosphorylation in Myocarditis Hearts

**FIGURE 2:**
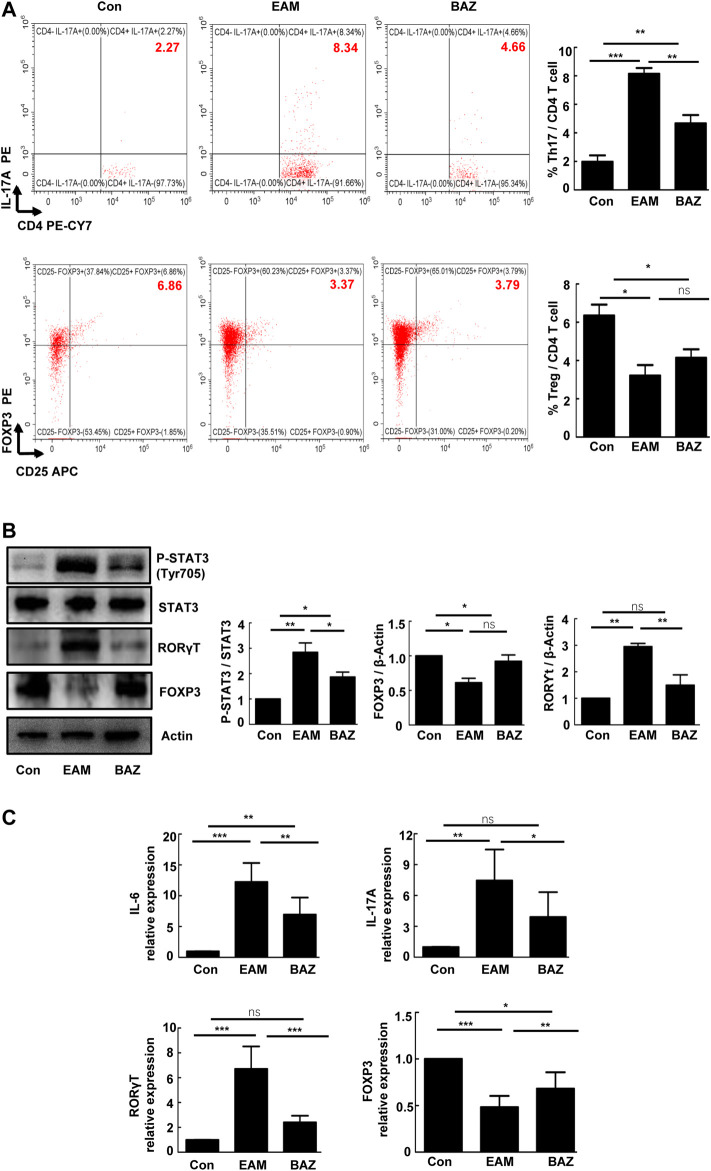
Bazedoxifene reduced Th17 cell infiltration and inhibited STAT3 phosphorylation in EAM hearts at day 21 **(A)**. The percentages of Th17 cells and Treg cells in isolated heart-infiltrating cells of each group (*n* = 6) **(B)**. Representative images of Western blots for RORγt, FOXP3 p-STAT3, and STAT3 protein from heart-infiltrating cells (*n* = 6). **(C)**. The mRNA expression levels of RORγt, Foxp3, and inflammatory factors (IL-6 and IL-17A) in isolated heart-infiltrating cells of the indicated groups (*n* = 5). *p < 0.05, **p < 0.01, ***p < 0.001.

Previous studies have demonstrated that Th17/Treg imbalance is associated with the development of EAM (Yamashita et al., 2011). In this study, Bazedoxifene treatment significantly decreased infiltration of Th17 cells and slightly increased the number of Treg cells in EAM hearts (Figure 2A). Expression of RORγt (a critical transcriptional factor for Th17 differentiation) was enhanced in heart-infiltrating lymphocytes in EAM mice, but reduced by Bazedoxifene treatment. Correspondingly, the reduced expression of FOXP3 (a maker of Treg cells), characteristic of heart-infiltrating lymphocytes in EAM mice, was partially restored by Bazedoxifene (Figure 2B). Bazedoxifene also inhibited EAM-induced STAT3 phosphorylation (p-STAT3) in heart-infiltrating lymphocytes. In addition, Bazedoxifene could partially normalize mRNA expression of IL-6 and IL-17A in heart tissues of EAM mice, consistent with the levels of RORγt mRNA expression in heart-infiltrating lymphocytes (Figure 2C).

### Bazedoxifene Attenuated the EAM-Induced Differentiation and Th17 Cell Function

**FIGURE 3:**
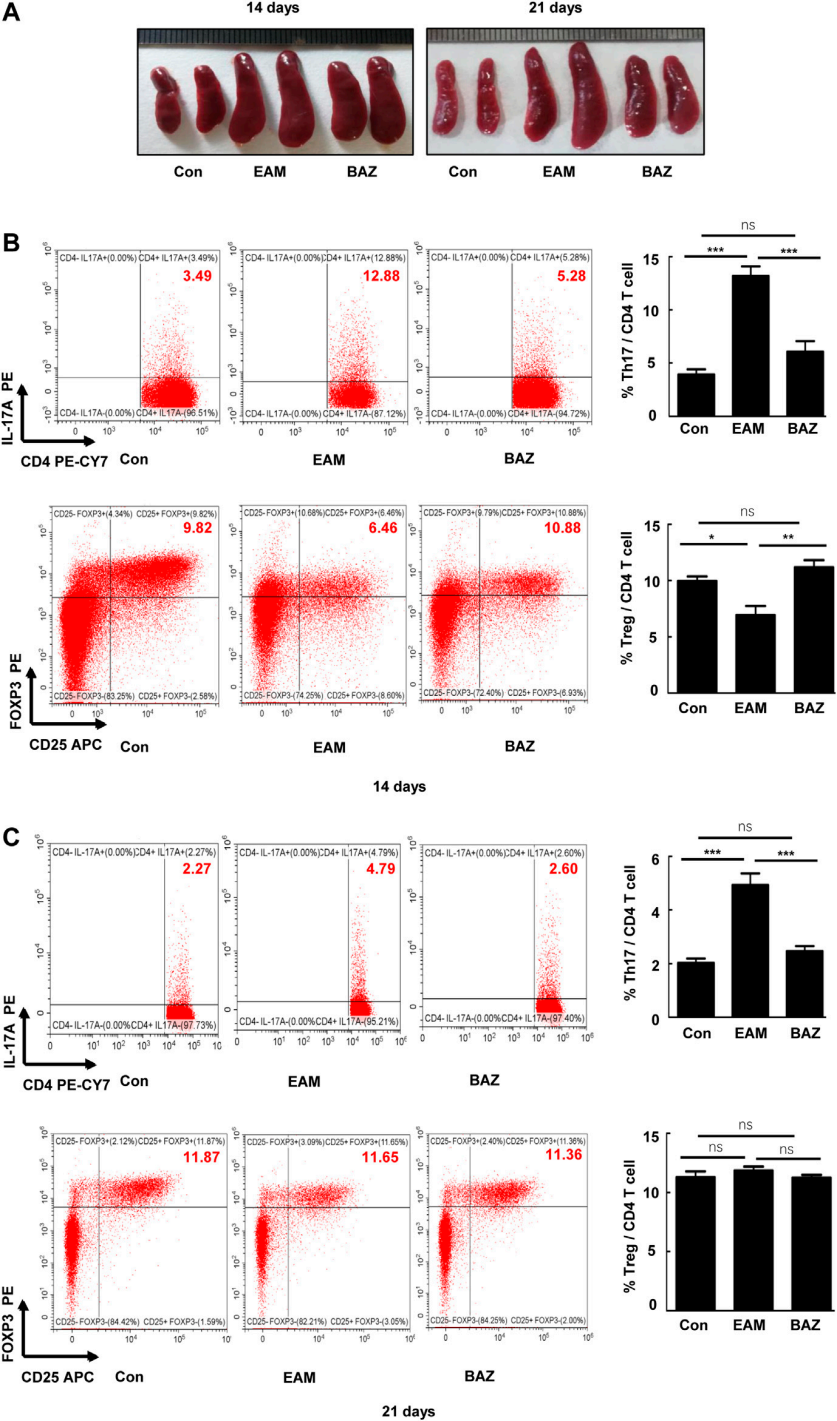
Bazedoxifene attenuated the EAM-induced differentiation of Th17 and Treg cells in EAM splenic CD4^+^ T cells at 14 and 21 days **(A)**. Representative spleen images of each group at days 14 and 21, respectively. The percentage of Th17 and Treg cells from spleen were determined at days 14 **(B)** and 21 **(C)** by flow cytometry. Representative FACS pictures of each group are shown. Averages are presented graphically on the right. *p < 0.05, **p < 0.01, ***p < 0.001.

**FIGURE 4:**
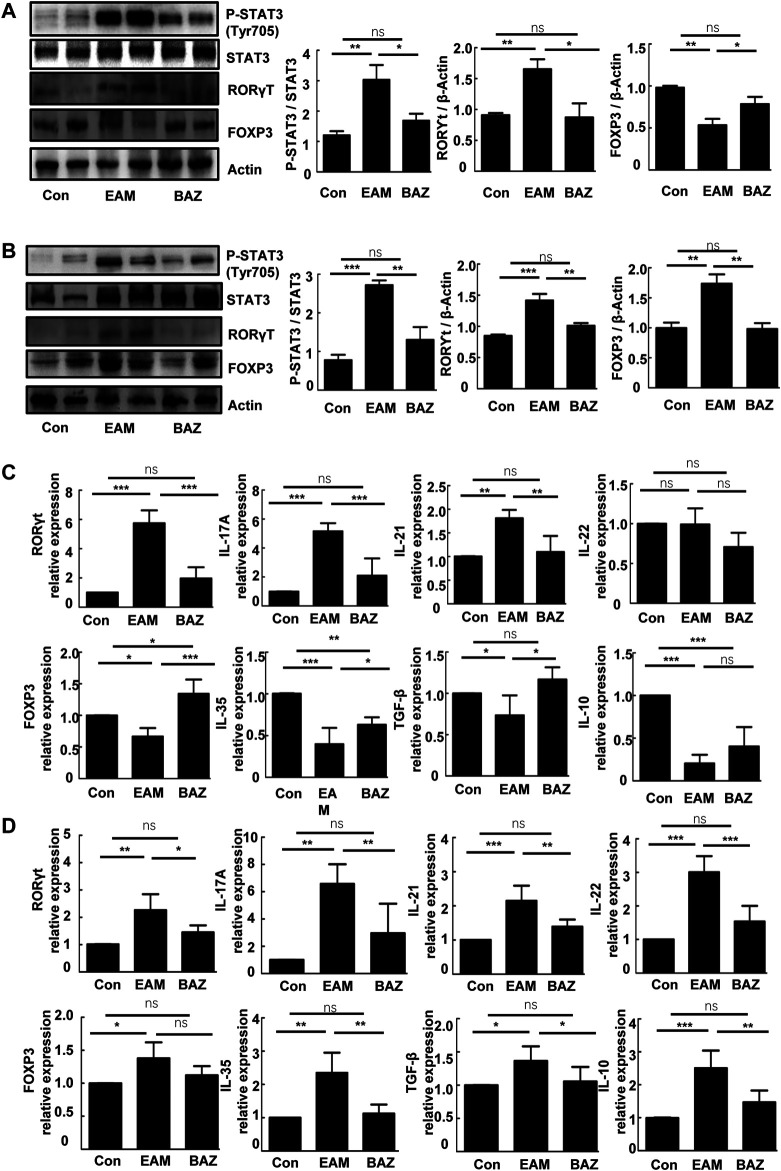
Bazedoxifene affected expression of the Th17/Treg cell ratio and related inflammatory factors in EAM splenic CD4^+^ T cells at 14 and 21 days. Western blots are shown for p-STAT3 (Tyr 705), STAT3, RORγt, and Foxp3 protein in splenic CD4^+^ T cells of each group at days 14 **(A)** and 21 **(B)**. mRNA expression is shown for Th17 cell-related factors and Treg cell-related factors in splenic CD4^+^ T cells from each group at days 14 **(C)** and 21 **(D)** (*n* = 4 per control group). *p < 0.05, **p < 0.01, ***p < 0.001.

Previous studies demonstrated that injection of control mice with the cardiac-specific peptide MyHC-α_614–629_ elicited an immune response that reached a maximum after 14 days, prior to obvious cardiac remodeling or dysfunction (Bronietzki et al., 2015). Spleens in untreated EAM mice were enlarged macroscopically (Figure 3A). In addition, Bazedoxifene could inhibit the EAM-induced increase in CD4^+^ IL-17A + T cells as a percentage of total splenic CD4^+^ T cells at day 14 and 21. The percentage of CD4^+^ CD25 ^+^ FOXP3+ Treg cells was reduced at day 14 in EAM mice, but was increased by Bazedoxifene treatment. There were no significant changes in the percentage of Tregs among the three groups at 21 days (Figures 3B,C). Bazedoxifene could inhibit STAT3 activation and RORγt expression in splenic CD4^+^ T cells (Figures 4A,B). Additionally, at day 14 or 21, expression of Th17-related proinflammatory factors, including IL-17A, IL-21, and IL-22, was up-regulated in EAM mice vs. controls, which was abolished by Bazedoxifenethe treatment (Figures 4C,D). Hence, Bazedoxifene could inhibit Th17 cell differentiation and function. In addition, expression of Treg-related anti-inflammatory factors in splenic CD4^+^ T cells, including IL-35, TGF-β, and IL-10, was reduced at day 14 and slightly increased at day 21 in EAM mice (Figures 4C,D). Expression of these factors was up-regulated at day 14 and down-regulated slightly at day 21 after Bazedoxifene treatment. The results were consistent with Foxp3 expression in splenic CD4^+^ T cells at days 14 and 21 (Figures 4A,B). In addition, the previous study suggested that P-STAT1, P-STAT 3, and P-STAT four could be activated in splenic CD4^+^ T cells. The specific JAK inhibitor AG490 inhibited STAT 1, 3, and four phosphorylation (Liu et al., 2016). The expression level of P-STAT1 and P-STAT4 were increased in EAM, which could not be inhibited by Bazedoxifene (Supplementary Figure S3). EAM might involve different kinds of STAT. But Bazedoxifene had no significant effect on other STATs, except for STAT3.

### Bazedoxifene Inhibited Th17 Cell Differentiation and Proliferation *in vitro*

**FIGURE 5:**
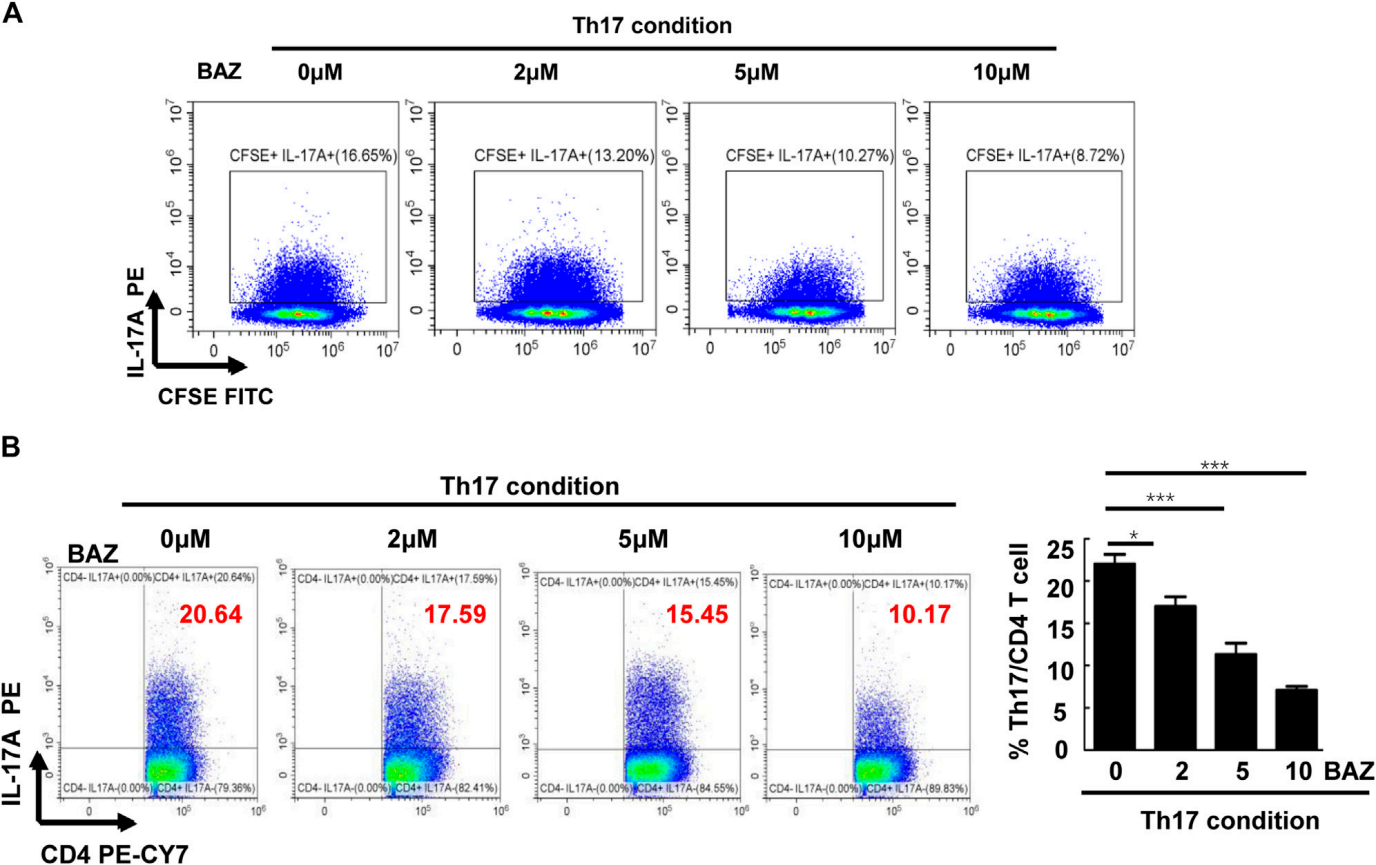
Bazedoxifene inhibited Th17 cell differentiation and proliferation *in vitro*. **(A)** Naïve T cells were labeled with CFSE and generated under Th17-polarizing conditions with different Bazedoxifene concentrations. **(B)** Naïve T cells sorted from healthy mice were cultured under Th17-polarizing conditions in the presence or absence of Bazedoxifene treatment for 72 h (*n* = 3 in each group). *p < 0.05, **p < 0.01, ***p < 0.001.

To further investigate whether Bazedoxifene affected CD4^+^ Th17 cell differentiation and proliferation *in vitro*, CD4^+^ T cells from healthy mice splenocytes were sorted and cultured in Th17-polarizing conditions. FACS analysis showed that Bazedoxifene could reduce the percentage of CD4^+^ IL-17A + Th17 cells (Figure 5B). Moreover, Bazedoxifene treatment effectively inhibited proliferation of Th17 cells (Figure 5A).

### Bazedoxifene Regulated Autophagy in Polarized Th17 Cells

**FIGURE 6:**
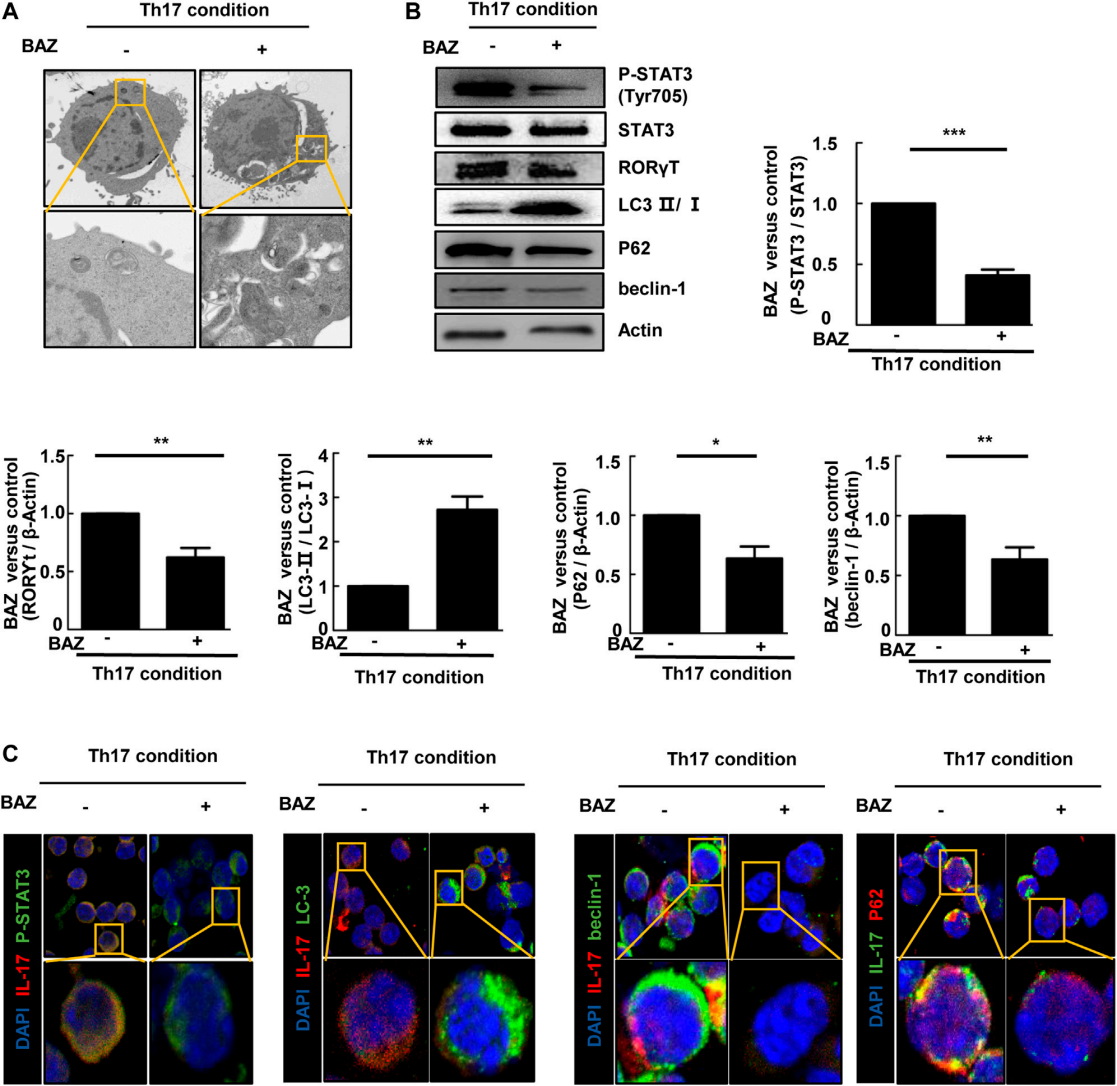
Bazedoxifene regulated autophagy in polarized Th17 cells **(A)**. Representative TEMs revealing the ultrastructure of CD4^+^ T cells cultured under Th17-polarizing conditions with or without Bazedoxifene treatment **(B)**. p-STAT3, STAT3, RORγt, LC3-Ⅱ/LC3-Ⅰ, P62, and beclin-1 protein levels were analyzed by western blotting **(C)**. Isolated naïve CD4^+^ T cells were cultured under Th17-polarizing condition in the presence or absence of Bazedoxifene. The cells were stained with anti-p-STAT3, anti-IL-17, anti-LC3, anti-beclin-1, and anti-P62 antibodies, and visualized using confocal microscopy. *p < 0.05, **p < 0.01, ***p < 0.001.

Autophagy is considered to be critically important for T cell differentiation into Th1 and Treg cells (Kodama et al., 1994). However, the importance of autophagy for Th17 cell development is unclear. TEM showed that double-membrane autophagic vesicles (autophagosomes), a marker of autophagy, were more prominent in naïve CD4^+^ T cells treated with Bazedoxifene under Th17-polarizing conditions (Figure 6A). Our results showed that the ratio of LC3-Ⅱ/LC3-Ⅰ in Bazedoxifene-treated Th17 cells was elevated compared with untreated Th17 cells, with a corresponding decrease in P62. Beclin-1 was down-regulated in Bazedoxifene-treated Th17 cells compared with the Th17 group (Figure 6B). The data above indicated that Bazedoxifene could enhance maturation of autophagosomes, which exist intrinsically during Th17 differentiation. Treatment with Bazedoxifene suppressed STAT3 activation and RORγt expression under Th17-polarizing conditions (Figure 6B). As shown in Figure 6C, co-staining of diffuse STAT3 phosphorylation and IL-17A, mainly located in the cytoplasm under Th17-polarizing conditions, was diminished upon Bazedoxifene treatment. Compared with Th17-polarizing conditions, the increased LC3 puncta indicated that more autophagosomes were induced, consistent with reduced P62 and beclin-1 puncta in the cytoplasm.

## Discussion

The EAM model in mice simulates the pathogenesis of myocarditis. Before day 14, it mainly manifests as a strong immune response in the spleen, including recognition of the α-MyHC peptide by CD4^+^ T cells and differentiation of detrimental CD4^+^ Th17 cells. The peak of cardiac inflammation occurs between days 14 and 21, and is characterized by the migration and infiltration of heart-specific CD4^+^ T cells from the spleen into the myocardium (Yan et al., 2016). Th17 cells have been proposed to play a prominent role in establishing inflammatory processes, while Treg cells act to restrain excessive immune responses. A proliferative or functional imbalance of the Th17/Treg ratio contributes to the pathogenesis of EAM (Quah et al., 2007). In this study, our data demonstrated an imbalance of Th17/Treg in splenic CD4^+^ T cells in the immune response at day 14 and heart-infiltrating CD4^+^ T cells at day 21. Bazedoxifene could suppress EAM via reciprocal regulation of Th17 cells and Treg cells at day 14, but the percentage of Treg cells was not reduced in EAM splenic CD4^+^ T cells at day 21. This may be due to inhibition of the IL-6/STAT3 signaling pathway by Bazedoxifene. Inhibition of STAT3 activity and expression of its downstream target, RORγt, decreased upon Bazedoxifene treatment. However, Foxp3, a key marker of Treg cell development, is not a direct downstream target of STAT3, which may partially explain why proliferation of Tregs in EAM was not significantly reduced by Bazedoxifene. Although the proportion of Treg cells did not continue to increase during the different immune response phases of EAM, the percentage of Th17 cells among splenic CD4^+^ T cells and heart-infiltrating lymphocytes continued to decrease upon Bazedoxifene treatment. Inhibition of STAT3 activity by Bazedoxifene resulted in a reduction in RORγt expression in Th17-polarizing conditions *in vitro*. There was also an increase in Th17-related inflammatory factors, such as IL-17A, IL-21, and IL-22, which can induce myocardial injury and remodeling. The elevated levels of inflammatory factors in EAM mice at day 14 and 21 could be reduced by Bazedoxifene. Accordingly, myocardial fibrosis in EAM mice could be ameliorated by the Bazedoxifene treatment (Ye et al., 2017; Wang et al., 2018; Kumar et al., 2019). Myocarditis is the result of a combination of inflammatory cytokines, among which CD4^+^ Th17 cell-related cytokines play a major role in the disease. Bazedoxifene might be a promising treatment for EAM via regulating the Th17 immune response by inhibiting STAT3 activation.

Serum levels of IL-6 are elevated in the mouse model of EAM and in patients with acute myocarditis, which is associated with a poor prognosis. In physiological or pathological conditions, IL-6 production could be triggered by a wide range of stimuli, such as stress hormones, cytokines, and pathogen recognition from different kinds of cells (Dittrich et al., 1994). In some inflammatory cardiovascular diseases, expression of IL-6 is dependent on STAT3. Inhibition of STAT3 activation results in reducing the expression of IL-6 in myocardium or blood vessels (Shi et al., 2020; Yan et al., 2020). In autoimmune disease, dendritic cells play an indisputable role in instructing the polarization of CD4^+^ Th17 cells in immune response through generation of cytokines such as IL-6, IL-23, and IL-1β (Coutant and Miossec, 2016). Reduced proportion of CD11c + dendritic cells and ability to produce Th17-polarizing cytokines are involved in the treatment of EAM (Yan et al., 2016). Inhibiting dendritic cell–derived IL-6 production could suppress Th17 differentiation in experimental autoimmune encephalomyelitis and experimental autoimmune myocarditis (Yan et al., 2016; Yang et al., 2018). And p-STAT3 signaling is necessary for dendritic cells’ differentiation by IL-6 binding to the IL-6 receptor (Park et al., 2004). Exposure of immature monocyte-derived dendritic cells to HIV-1 results in the production of IL-6 via mitogen-activated protein kinase (MAPK)/NF-κB pathways (Del Cornò et al., 2014). IL-6 in turn activates STAT3 by an autocrine loop. In our study, Bazedoxifene led to a decreased frequency of splenic CD11c + dendritic cells in EAM mice (Supplementary Figure S1A). Additionally, the circulating levels of IL-23 and IL-6 were reduced in mice with Bazedoxifene treatment (Supplementary Figure S1B). Therefore, Bazedoxifene could suppress the ratio of dendritic cells in mice splenic cells, as well as the secretion of inflammatory cytokines. This may provide an explanation for the decreased IL-6 production in our results.

Previous research has shown that the IL-6/STAT3/RORγt signaling pathway is essential in Th17 cell differentiation and function in EAM (Bronietzki et al., 2015). The blockade of the IL-6 receptors by antibodies or inhibition of STAT3 activation by small-molecule inhibitors prevented the development of EAM, accompanied by reduced expression of RORγt. However, these results are difficult to translate into clinical practice due to uncertain toxicity and bio-availability. Bazedoxifene has undergone clinical trials and been approved by the FDA, which provides a basis for investigating its use for other diseases. In our results, Bazedoxifene could inhibit IL-6 induced STAT3 phosphorylation in CD4^+^ T cells *in vivo* and vitro. Additionally, estradiol and stattic were added to clarify the possible mechanisms under Th17 cell differentiation with treatment of Bazedoxifene. In Supplementary Figure S6, compared with Th17 polarization, BAZ and Stattic treatment could suppress Th17 cells’ proliferation and differentiation to varying degrees. However, estradiol treatment had little inhibiting effect on Th17 cell differentiation in polarized conditions. Bazedoxifene has been reported as a novel inhibitor of IL-6/GP130 protein−protein interactions (PPIs) using multiple ligand simultaneous docking (MLSD) and drug repositioning approaches (Li et al., 2014). Induction of STAT3 phosphorylation by IL-6 could be inhibited with the treatment of Bazedoxifene in Hep3B cells which are considered as estrogen receptor-negative (Ma et al., 2019). In addition, Bazedoxifene has no effect on T lymphopoiesis and T cell dependent inflammation in a delayed type hypersensitivity model, independent from estrogen receptors (Bernardi et al., 2015). Hence, we speculate the suppressive effect on the constitutive expression of p-STAT3 by Bazedoxifene might not be estrogen receptor-dependent.

Autophagy involves the selective degradation of cellular components. Recent studies have provided evidence that autophagy also plays an important role in autoimmune disease by modulating the function of CD4^+^ T cell (Kreuzaler et al., 2011). However, few studies have addressed the relationship between autophagy and Th17 cell differentiation. Growing evidence suggests that the STAT3 signaling pathway affects autophagy in various ways (Pensa et al., 2014). To clarify this issue more clearly, we tried Stattic as a positive control. Stattic is a kind of STAT3 inhibitor. In Supplementary Figure S2, inhibition of p-STAT3 by Bazedoxifene and Stattic was accompanied by an increase in ratio of LC3-II/LC3-I in polarized Th17 cells. For other markers of autophagy, the expression of P62 and beclin-1 were lower in BAZ group than in Stattic group. Moreover, Th17 cell differentiation was suppressed with treatment of BAZ and Stattic, as the protein level of RORγt was decreased. STAT3 is a transcription factor that mediates cellular responses to a variety of cytokines and growth factors, of which IL-6 plays an important role in immunity (Yu et al., 2009). In our results, Th17 differentiation was reduced and autophagy was promoted via partial inhibition of STAT3 by the IL-6 signaling pathway in BAZ group, the effect of which was more obviously produced via total inhibition of STAT3 by Stattic. This provides evidence that STAT3 phosphorylation induces autophagy. Although the molecular mechanism between autophagy and STAT3 activation in Th17 cell differentiation and function is not fully understood, our results may provide new clues for exploring relationships between Th17 cell autophagy and anti-inflammatory signaling pathways in EAM. The results of our study suggest that Bazedoxifene may be appropriate for a range of clinical indications in cardiovascular disease and may serve as a template for developing novel agents to treat autoimmune myocarditis.

## Conclusion

We have demonstrated that Bazedoxifene can play a protective role in EAM, as it ameliorates cardiac inflammation, reduces myocardial injury, and improves cardiac function by inhibiting Th17 cell development *in vivo*. Bazedoxifene inhibited Th17 cell differentiation and function and regulated autophagy in Th17 cells *in vitro*. Elucidation of the role of STAT3 in autophagy may provide a comprehensive understanding of Th17 development. Our study suggests a novel therapeutic strategy for reducing inflammation and myocardial injury in EAM via inhibiting Th17 cell differentiation and function.

## Data Availability

The original contributions presented in the study are included in the article/Supplementary Material, further inquiries can be directed to the corresponding author.

## Supplementary Material

Supplementary Figure 1 | The effect of Bazedoxifene on dendritic cells about the differentiation and secreting Th17-polarizing cytokines in EAM mice. **(A)** The proportion of CD11c+ dendritic cells among splenocytes in each group. Representative FACS pictures of each group are shown. Averages are presented graphically on the right. **(B)** The serum levels of Th17-polarizing cytokines (IL-6 and IL-23) and Th17-secrecting cytokine (IL-17A) (n = 5). **P < 0.05, **P < 0.01, ***P < 0.001*.

Supplementary Figure 2 | Inhibition of STAT3 phosphorylation modulated autophagy in polarized Th17 cells. To determine whether the regulatory property of Bazedoxifene on Th17 differentiation affected the induction of autophagy, splenic CD4+ T cells from Balb/c mice were cultured under conditions inducing Th17 differentiation in the presence or absence of Bazedoxifene (5 µM) or Stattic (5 µM) for 72 hours. P-STAT3, STAT3, RORγt, LC3-II/LC3-I, P62, and beclin-1 protein levels were analyzed by western blotting. **P < 0.05, **P < 0.01, ***P < 0.001*.

Supplementary Figure 3 | Changes of phosphorylated proteins of STAT 1 and 4 signaling pathway. The expression level of p-STAT1, STAT1, p-STAT4 and STAT4 was detected in each group by western blotting. **P < 0.05, **P < 0.01, ***P < 0.001*.

Supplementary Figure 4 | The body weight of mice in control, EAM and BAZ group. **P* < 0.05, ***P* < 0.01, ****P* < 0.001

Supplementary Figure 5 | Measurements of blood pressure in mice at day 21 using the NIBP system by cuffing the tails. The bar graph shows a quantitative analysis of systolic BP (SBP), mean BP (MBP), and diastolic BP (DBP).

Supplementary Figure 6 | The effect of inhibiting p-STAT3 and activating estrogen receptor signaling pathway on Th17 cell differentiation and proliferation in vitro. Naïve T cells sorted from Balb/c mice were cultured under Th17-polarizing conditions in the presence or absence of Bazedoxifene (5 µM), estradiol (E2, 1 nmol/l) or Stattic (5 µM) for 72 hours. Averages are presented graphically on the right. **P < 0.05, **P < 0.01, ***P < 0.001*.
