# Supplementary figures and images for: Bazedoxifene Regulates Th17 Immune Response to Ameliorate Experimental Autoimmune myocarditis via Inhibition of STAT3 Activation
Source: Front Pharmacol. 2021 Feb 10;11:613160. doi: 10.3389/fphar.2020.613160 (PMC7903338; doi:10.3389/fphar.2020.613160)

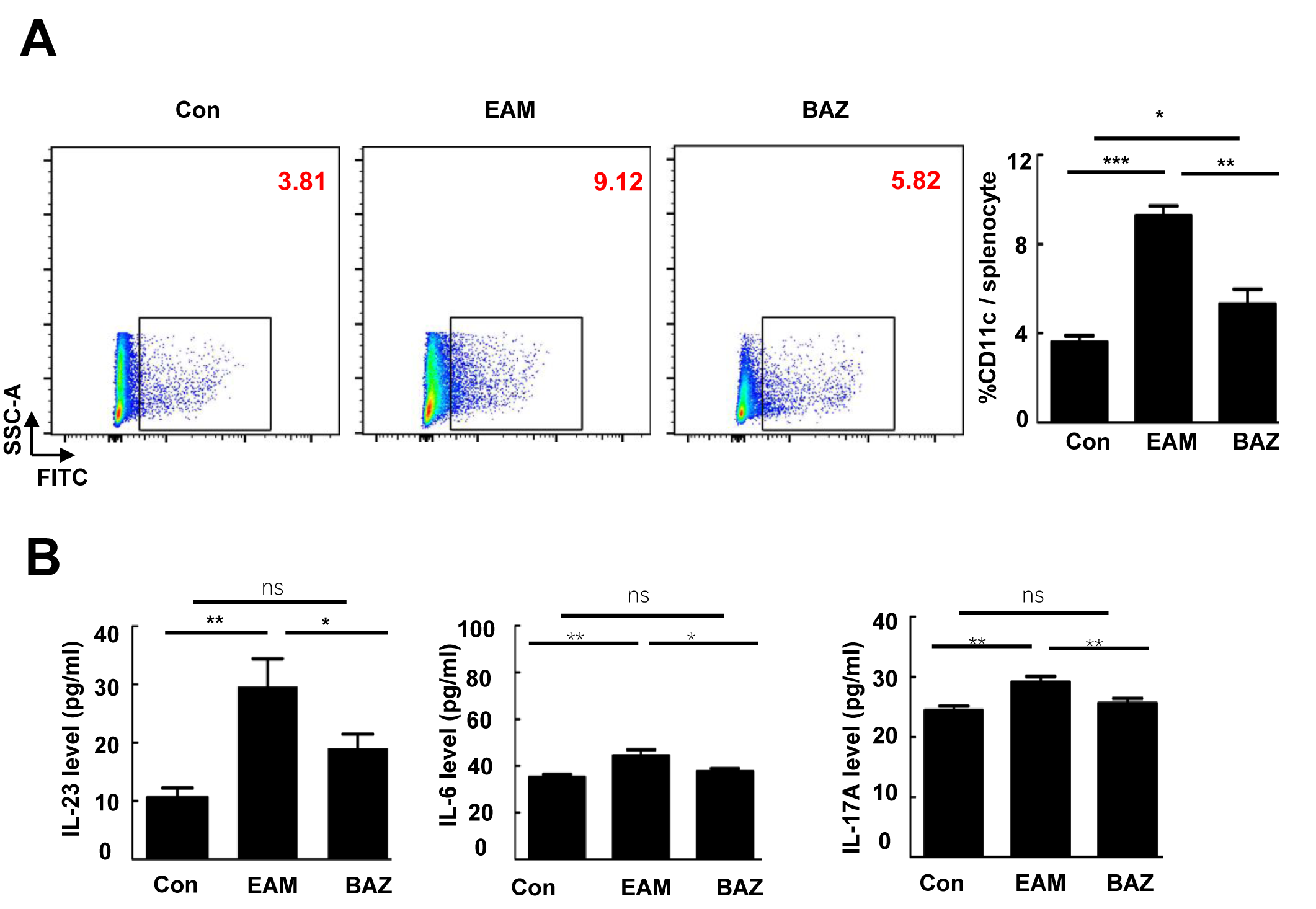

Supplement: Supplementary file 1 [file image1.tif]

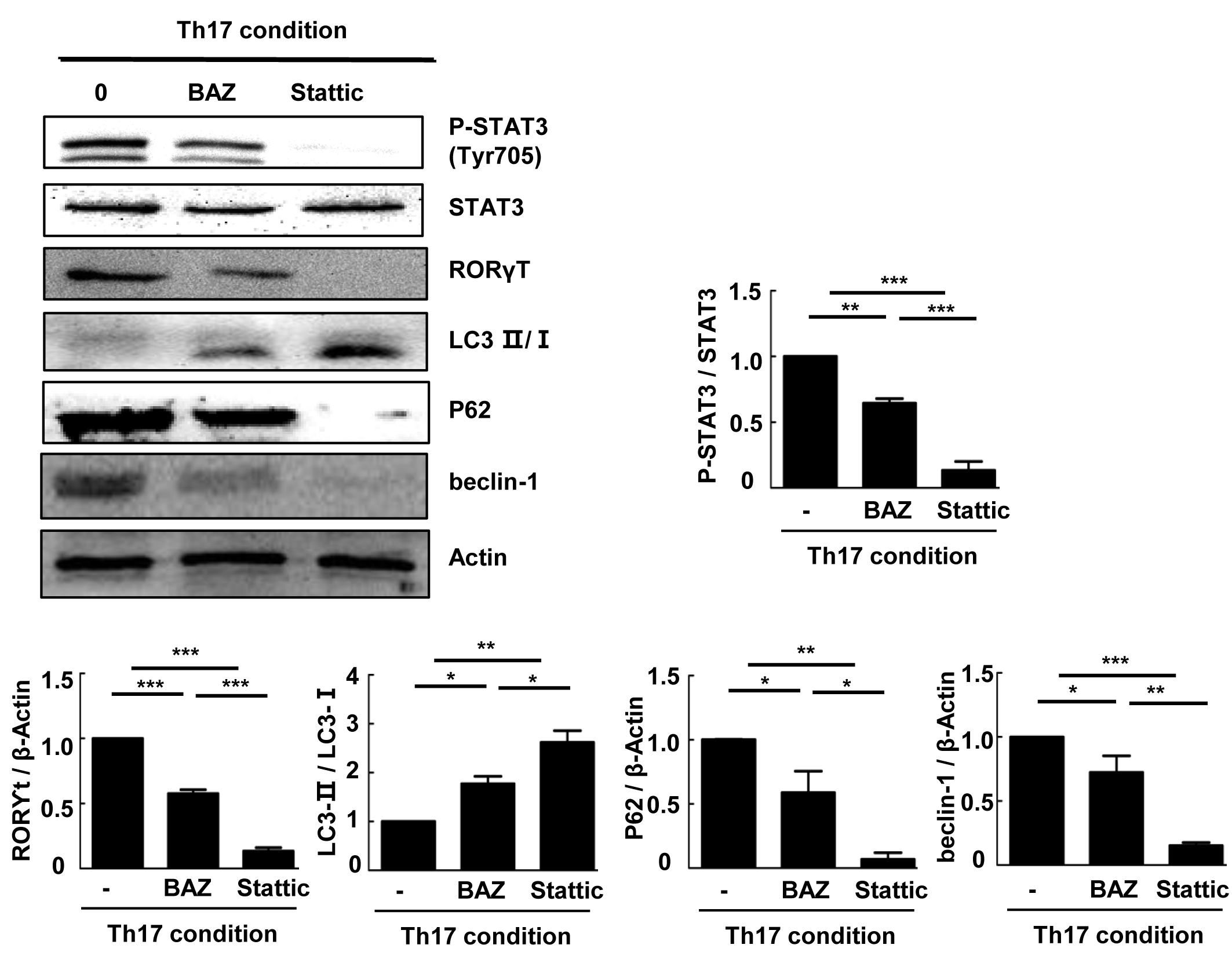

Supplement: Supplementary file 2 [file image2.tif]

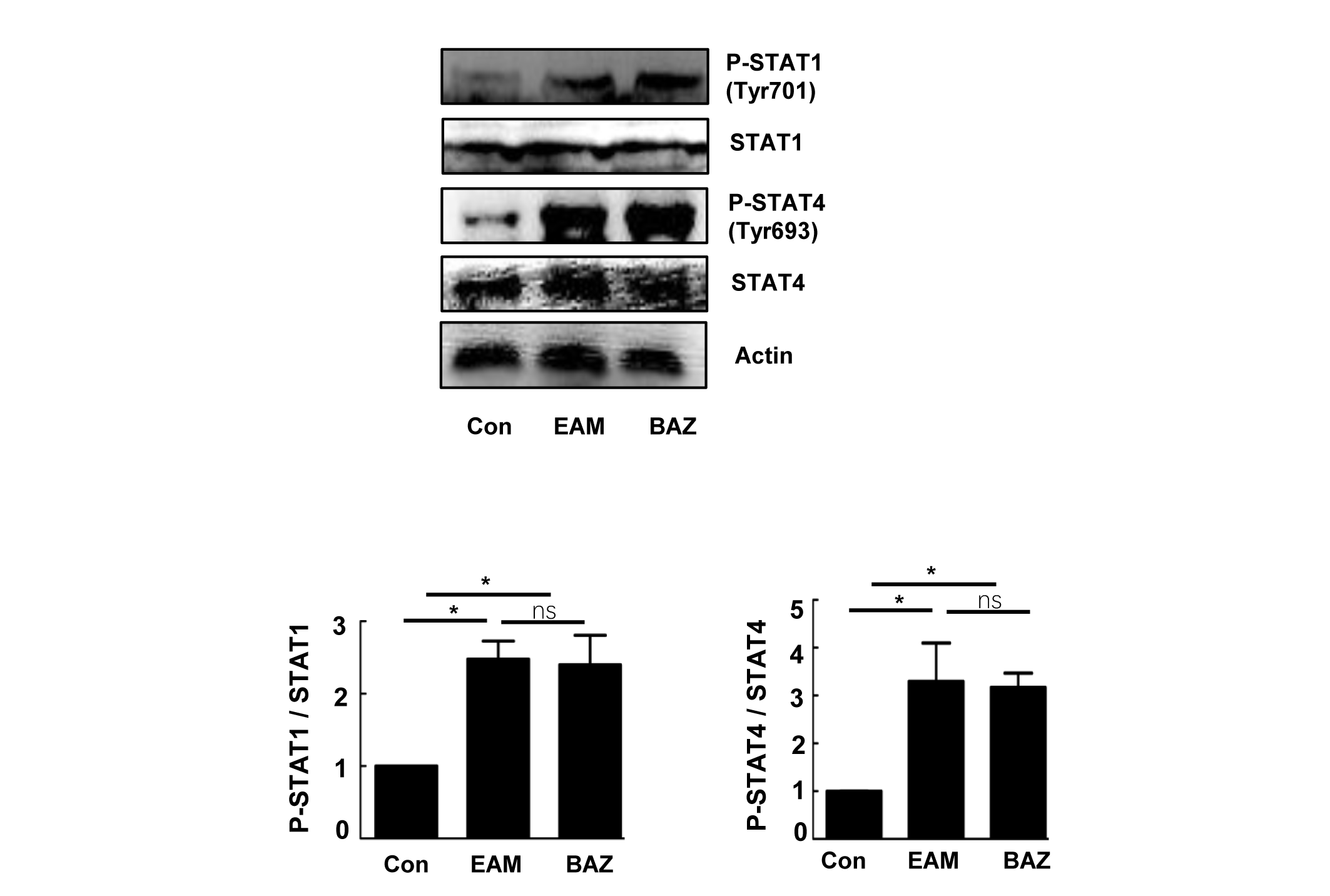

Supplement: Supplementary file 3 [file image3.tif]

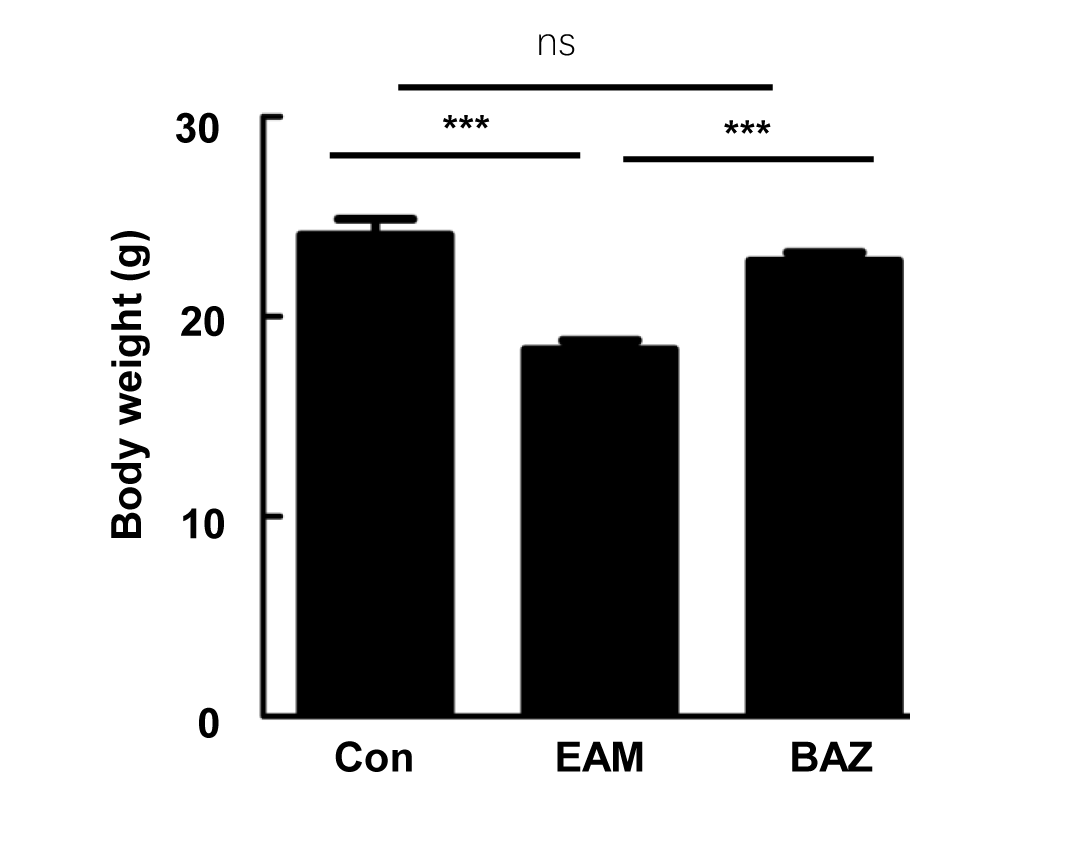

Supplement: Supplementary file 4 [file image4.tif]

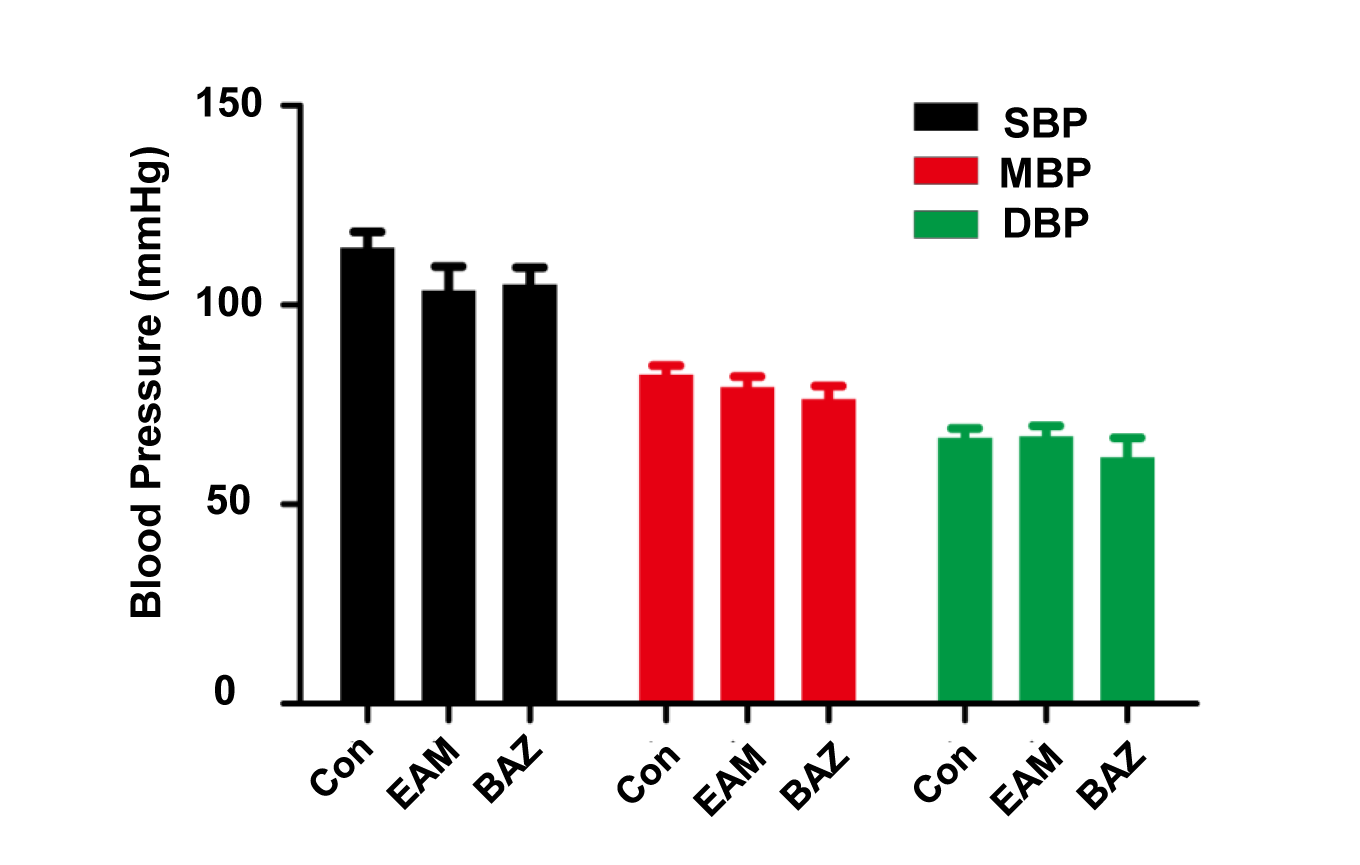

Supplement: Supplementary file 5 [file image5.tif]

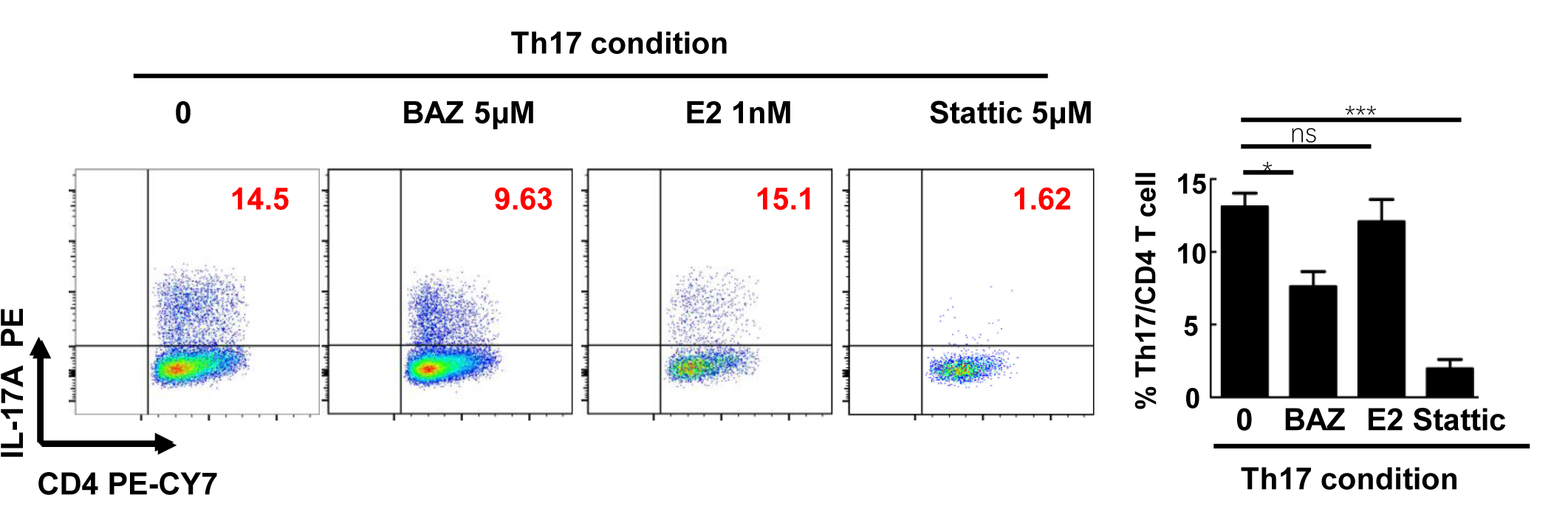

Supplement: Supplementary file 6 [file image6.tif]
