# Supplementary material for: Bazedoxifene Regulates Th17 Immune Response to Ameliorate Experimental Autoimmune myocarditis via Inhibition of STAT3 Activation
Source: Front Pharmacol. 2021 Feb 10;11:613160. doi: 10.3389/fphar.2020.613160 (PMC7903338; doi:10.3389/fphar.2020.613160)
Supplement: Supplementary file 7 [file Table2.DOCX]

**Supplementary Figure 1.** The effect of Bazedoxifene on dendritic cells about the differentiation and secreting Th17-polarizing cytokines in EAM mice. (A) The proportion of CD11c+ dendritic cells among splenocytes in each group. Representative FACS pictures of each group are shown. Averages are presented graphically on the right. (B) The serum levels of Th17-polarizing cytokines (IL-6 and IL-23) and Th17-secrecting cytokine (IL-17A) (n=5). **P < 0.05, **P < 0.01, ***P < 0.001*

**Supplementary Figure 2.** Inhibition of STAT3 phosphorylation modulated autophagy in polarized Th17 cells. To determine whether the regulatory property of Bazedoxifene on Th17 differentiation affected the induction of autophagy, splenic CD4+ T cells from Balb/c mice were cultured under conditions inducing Th17 differentiation in the presence or absence of Bazedoxifene (5μM) or Stattic (5μM) for 72 hours. P-STAT3, STAT3, RORγt, LC3-Ⅱ/LC3-Ⅰ, P62, and beclin-1 protein levels were analyzed by western blotting. **P < 0.05, **P < 0.01, ***P < 0.001*

**Supplementary Figure 3.** Changes of phosphorylated proteins of STAT 1 and 4 signaling pathway. The expression level of p-STAT1, STAT1, p-STAT4 and STAT4 was detected in each group by western blotting. **P < 0.05, **P < 0.01, ***P < 0.001*

**Supplementary Figure 4.** The body weight of mice in control, EAM and BAZ group. **P < 0.05, **P < 0.01, ***P < 0.001*

**Supplementary Figure 5.** Measurements of blood pressure in mice at day 21 using the NIBP system by cuffing the tails. The bar graph shows a quantitative analysis of systolic BP (SBP), mean BP (MBP), and diastolic BP (DBP).

**Supplementary Figure 6.** The effect of inhibiting p-STAT3 and activating estrogen receptor signaling pathway on Th17 cell differentiation and proliferation in vitro. Naïve T cells sorted from Balb/c mice were cultured under Th17-polarizing conditions in the presence or absence of Bazedoxifene (5μM), estradiol (E2, 1 nmol/l) or Stattic (5μM) for 72 hours. Averages are presented graphically on the right. **P < 0.05, **P < 0.01, ***P < 0.001*
